# Polyamine Derived Photosensitizer: A Novel Approach for Photodynamic Therapy of Cancer

**DOI:** 10.3390/molecules29174277

**Published:** 2024-09-09

**Authors:** Hao Deng, Ke Xie, Liling Hu, Xiaowen Liu, Qingyun Li, Donghui Xie, Fengyi Xiang, Wei Liu, Weihong Zheng, Shuzhang Xiao, Jun Zheng, Xiao Tan

**Affiliations:** 1 Hubei Key Laboratory of Tumor Microenvironment and Immunotherapy, College of Basic Medical Science, China Three Gorges University, Yichang 443002, China; patience1231@hotmail.com (H.D.);; 2 The First College of Clinical Medical Science, China Three Gorges University, Yichang 443003, Chinazhengjun1995@163.com (J.Z.); 3 College of Biological and Pharmaceutical Sciences, China Three Gorges University, Yichang 443002, China

**Keywords:** photosensitizer, polyamine transport system, cancer, photodynamic therapy

## Abstract

Polyamines play a pivotal role in cancer cell proliferation. The excessive polyamine requirement of these malignancies is satisfied through heightened biosynthesis and augmented extracellular uptake via the polyamine transport system (PTS) present on the cell membrane. Meanwhile, photodynamic therapy (PDT) emerges as an effective anti-cancer treatment devoid of drug resistance. Recognizing these intricacies, our study devised a novel polyamine-derived photosensitizer (PS) for targeted photodynamic treatment, focusing predominantly on pancreatic cancer cells. We synthesized and evaluated novel spermine-derived fluorescent probes (N2) and PS (N3), exhibiting selectivity towards pancreatic cancer cells via PTS. N3 showed minimal dark toxicity but significant phototoxicity upon irradiation, effectively causing cell death in vitro. A significant reduction in tumor volume was observed post-treatment with no pronounced dark toxicity using the pancreatic cancer CDX mouse model, affirming the therapeutic potential of N3. Overall, our findings introduce a promising new strategy for cancer treatment, highlighting the potential of polyamine-derived PSs in PDT.

## 1. Introduction

PDT is currently one of the most promising strategies in the treatment of various malignant tumors, including pancreatic [1,2,3], lung [4], stomach [1], esophageal [5], and bladder cancers [6,7]. The most important factor of PDT is the PS, a critical element that transfers energy to the surrounding oxygen under light excitation, thereby generating singlet oxygen (^1^O_2_) and reactive oxygen species (ROS) [8]. These compounds ultimately result in the death of tumor cells. Unlike traditional chemotherapy drugs, PDT has limited resistance from cancer cells and demonstrates excellent tolerability in tumor therapies. Therefore, the primary focus of PS research lies in improving its specificity for targeting tumor cells and reducing dark toxicity during clinical application [8,9,10].

Pancreatic cancer is a malignant tumor with a low resection rate [11], PDT was first reported in pancreatic cancer treatment in the 1980s by Holyoke [12,13]. Numerous clinical trials investigating the effectiveness of PDT in treating pancreatic cancer have since been conducted [14,15,16,17], demonstrating significant potential in inhibiting tumor growth and extending patient survival. However, the PSs currently employed in clinical PDT for pancreatic cancer often lead to severe side effects, such as duodenal perforation and liver injury [11]. These adverse reactions are largely attributable to the high doses required to achieve effective therapeutic outcomes.

PTS is a membrane transport mechanism responsible for the cellular uptake of polyamines. Existing research has provided partial elucidation of the molecular mechanisms underlying the PTS [18,19]. It’s known that an upregulated PTS is crucial for maintaining high intracellular levels of polyamine in many cancer cells including pancreatic cancer cells [20]. Numerous studies suggest that the PTS can transfer both natural polyamines and polyamine derivatives into cancer cells, leading to the hypothesis that polyamine derivatives of cytotoxic drugs could selectively target cancer cells, thereby reducing overall toxicity [21,22]. Polyamines, which include putrescine, spermine, and spermidine, are central to this process. Delcros posited that the characteristics of the polyamine moiety, as well as the increase in the number of nitrogen centers are significant determinants of PTS affinity [23]. Spermine, in particular, is most effective at targeting the PTS for cellular entry, influencing the characteristics of the polyamine moiety and playing a crucial role in PTS affinity [24]. Existing studies have reported the synthesis of polyamine derivatives targeting the PTS, primarily for the creation of fluorescent probes to analyze the molecular mechanisms of PTS and their application in PDT. These investigations predominantly elucidate the potential application value of polyamine derivatives from a molecular mechanism and cellular-level experimental perspective. Thus, using pancreatic cancer as a case in point due to its high polyamine requirements, our study proposes the design and synthesis of spermine derivatives for use in PDT.

BODIPY derivatives, PSs carrying heavy atoms like iodine within their core structure [25] are extensively used in PDT due to their high extinction coefficient, exceptional photothermal stability, and flexible chemical structure [9,26,27]. BODIPY-2I, a derivative synthesized by Yogo et al., is commonly employed in PDT [28]. In recent years, the tumor-targeted modification of BODIPY derivatives has been actively pursued. In this study, we designed and synthesized a BODIPY-2I spermine derivative, named N3, for potential application in PDT targeting cancers with elevated polyamine demands, using pancreatic cancer as a representative case. However, due to N3′s high triplet quantum yield and weak fluorescence signal, tracking its presence within cells presents a significant challenge. To circumvent this issue, we initially synthesized a fluorescent spermine derivative (N2) to serve as a probe for labeling pancreatic cancer cells in our research.

## 2. Results

### 2.1. N2 Was Specifically Taken into PANC-1 Cells

**Scheme 1:**
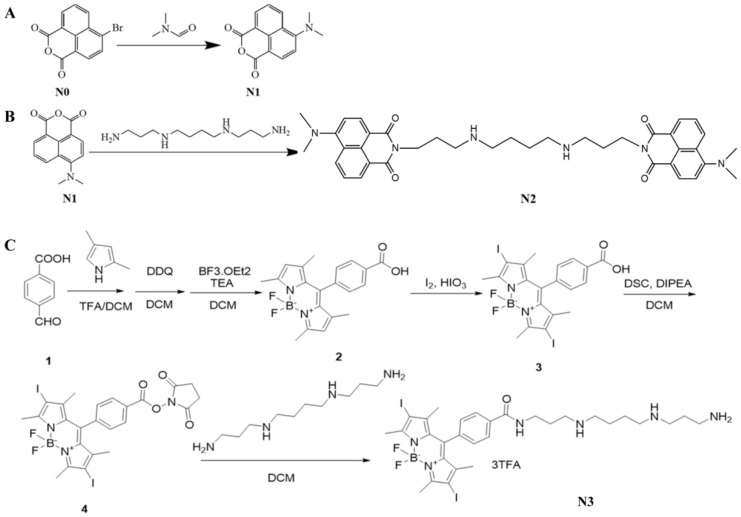
Synthetic routes of N1 (**A**), N2 (**B**), and N3 (**C**).

**Figure 1:**
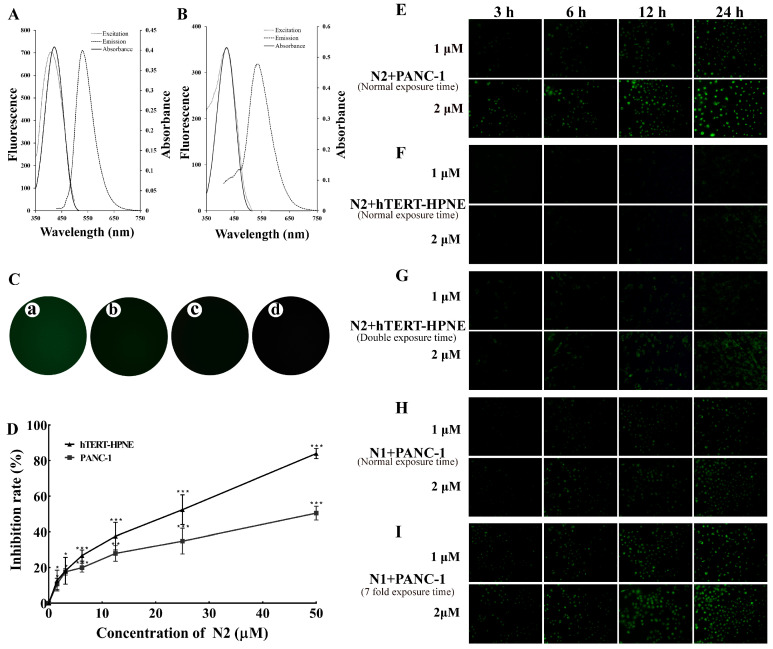
The uptake of N2 into PANC-1 and hTERT-HPNE cells. The excitation and emission wavelength of N1 (**A**) and N2 (**B**) were found at 420 nm and 530 nm; (**C**) Fluorescent property of N2 in different solvent: (a) ethanol; (b) DMSO; (c) DMEM and (d) water; (**D**) The toxicity of N2 in PANC-1 and hTERT-HPNE cells; (**E**) Fluorescence observed in PANC-1 cells after treated with 1 and 2 µM of N2 for 3, 6, 12 and 24 h, normal exposure time (200×); Fluorescence observed in hTERT-HPNE cells after treated with 1 and 2 µM of N2 for 3, 6, 12 and 24 h, normal exposure time (**F**), double exposure time (**G**) (200×); Fluorescence observed in PANC-1 cells after treated with 1 and 2 µM of N1 for 3, 6, 12 and 24 h, normal exposure time (**H**), 7 fold exposure time (**I**) (200×). * *p* < 0.05, ** *p* < 0.01, *** *p* < 0.001.

The spermine-derived fluorescent probe (N2) was synthesized using a simple method described in Scheme 1A,B. The purity and structure of the molecule were carefully analyzed using high-performance liquid chromatography (HPLC), mass spectrometry (MS), and nuclear magnetic resonance (NMR) (^1^H NMR and ^13^C NMR), which are shown in Supplementary Figure S1. The absorbance and excitation wavelengths of N1 and N2 were determined using UV spectrometry and found to be 420 nm. Additionally, the emission wavelength was identified using fluorescence spectrometry, which was found to be 530 nm for both compounds (Figure 1A,B).

Owing to the presence of a rotatable dimethylamino unit, probes N1 and N2 emit relatively weak fluorescence in hydrolytic solvents, but their fluorescence intensity increases significantly in hydrophobic environments which could restrict the rotation of the dimethylamino group (Figure 1C). As a result of this unique attribute, N2 can be employed to label cells for imaging purposes since the fluorescence of N2 is only detected when they are internalized into cells or comes into contact with the cell membrane. Furthermore, the spermine moiety of N2 does not affect the fluorescent properties of its fluorescence core N1, as suggested by the results above.

The cytotoxicity of N2 was first assessed in PANC-1 and hTERT-HPNE cells (Figure 1D). After incubating the cells with N2 for 24 h, it was found that N2 had a dose-dependent toxic effect on both PANC-1 and hTERT-HPNE cells. However, concentrations of N2 below 5 μM had very little toxicity on these cells. Therefore, in subsequent in vitro experiments, we aimed to limit the concentration of N2 to a maximum of 2 µM.

To evaluate the specific uptake ability of N2 by PANC-1 cells, we employed fluorescence microscopy to observe the uptake of N2 and N1 by both PANC-1 and hTERT-HPNE cells. The results indicated that, following treatment with 1 or 2 μM of N2 for 3 to 24 h, the fluorescence intensity of N2 taken up by PANC-1 cells increased in a time and dose-dependent manner (Figure 1E). However, the fluorescence of N2 taken up by hTERT-HPNE cells remained undetectable even after a 24-h incubation period (Figure 1F) and the fluorescence signal remained weak even when the exposure time was doubled (Figure 1G).

To further verify the specific uptake of N2 by PANC-1 cells, we used N1 as a control. After incubation of PANC-1 cells with N1 for 24 h, the fluorescence signal from N1 taken up by PANC-1 cells was hardly detectable (Figure 1H). When the exposure time was increased to seven-fold, the fluorescence signal of N1 could be faintly observed but was significantly interfered with by the background fluorescence (Figure 1I).

These results suggest that N2 can specifically accumulate in PANC-1 cells, and compared to N1, the spermine moiety of N2 plays a significant role in the uptake of N2 by PANC-1 cells.

### 2.2. N2 Was Taken into PANC-1 Cells via PTS

**Figure 2:**
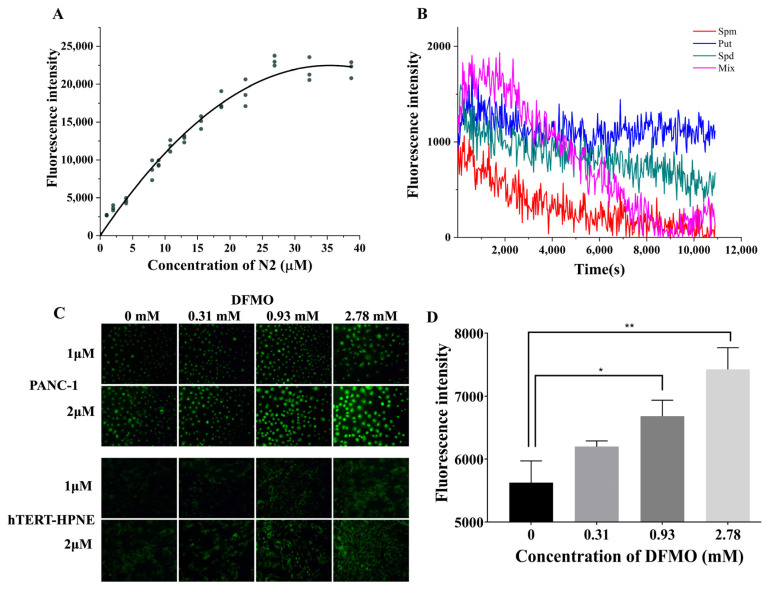
The uptake of N2 into PANC-1 cells relied on PTS. (**A**) The rate of uptake of N2 into PANC-1 cells increased by increasing N2 concentration until PTS saturation; (**B**) The uptake of N2 (2 µM) into PANC-1 cells was inhibited by competition of polyamines (10 µM) (Spm: spermine; Put: putrescine; Spd: spermidine; Mix: a mixture of putrescine, spermidine, and spermine); (**C**) Uptake of N2 (1 and 2 µM) observed in PANC-1 (normal exposure time) and hTERT-HPNE (5 fold exposure time) cells in presence of a different concentration of DFMO (0, 0.31, 0.93 and 2.78 mM) (200×); (**D**) Evaluation of uptake of N2 (2 µM) into PANC-1 using spectrofluorometry in presence of different concentration of DFMO (0, 0.31, 0.93 and 2.78 mM). DFMO promoted the uptake of N2 into PANC-1 cells. * *p* < 0.05, ** *p* < 0.01.

To investigate the mechanism of N2 uptake by pancreatic cancer cells, we treated PANC-1 cells with different concentrations of N2 over a short period and then analyzed the uptake rate of N2 in PANC-1 cells using flow cytometry (Figure 2A). The results revealed that as the concentration of N2 increased, the fluorescence intensity increased until a N2 concentration of 25μM was reached. Beyond this point, further increases in N2 concentration did not result in any additional increase in fluorescence. These findings suggest that the uptake rate of N2 by PANC-1 cells is concentration-dependent, reaching its maximum at a N2 concentration of 25 μM. This implies that the mechanism of N2 uptake by PANC-1 cells is not via free diffusion. Rather, it’s more likely that N2 enters PANC-1 cells through the PTS channels on the cell membrane in a passive diffusion manner.

Subsequently, we carried out a competition experiment to investigate whether the uptake of N2 by PANC-1 cells depends on the PTS on their cell membrane. To accomplish this, we used 10 µM of putrescine, spermidine, spermine, and their mixture as competitors, treating PANC-1 cells concurrently with N2 (2 µM). After accounting for the control group, i.e., excluding the fluorescence intensity changes in the group without competitors, we observed that putrescine, spermidine, spermine, and the polyamine mixture could inhibit the uptake of N2 by PANC-1 cells (Figure 2B). Among them, the inhibitory effect of the polyamine mixture was superior to that of using putrescine, spermidine, or spermine individually.

These results suggest that the uptake of N2 by PANC-1 cells depends on the PTS present on their cell membrane.

### 2.3. Low Concentration of DFMO Promoted Uptake of N2 into PANC-1 Cells

Difluoromethylornithine (DFMO) is an early clinically used cancer treatment drug that inhibits the activity of ornithine decarboxylase (ODC), reducing the biosynthesis of polyamines within cancer cells and thereby inhibiting their proliferation. However, this therapeutic effect is easily evaded by cancer cells by absorbing polyamines from the external environment via PTS.

Given DFMO’s inhibitory effect on intracellular polyamine biosynthesis, we hypothesized that low concentration of DFMO could enhance the activity of PTS by suppressing the cell’s internal polyamine synthesis, thereby increasing the uptake of exogenous polyamines or polyamine derivatives in PANC-1 cells. This would, in turn, enhance the accumulation of N2 within PANC-1 cells.

To investigate this, we first assessed the toxicity of DFMO on PANC-1 and hTERT-HPNE cells (Supplementary Figure S3). We found that when the concentration of DFMO was less than 10 mM, its toxicity to PANC-1 and hTERT-HPNE cells was extremely low. By using fluorescence microscopy and a spectrofluorometer to analyze the impact of DFMO on the absorption of N2 in PANC-1 and hTERT-HPNE cells (Figure 2C,D), we discovered that the fluorescent intensity of N2 taken up by PANC-1 cells increased with the concentration used of DFMO. However, the fluorescent intensity of N2 within hTERT-HPNE cells did not change significantly (up to 5 times the exposure time). This finding suggests that the inhibition of polyamine synthesis in PANC-1 cells promotes their uptake of N2 through PTS but this is not the case in hTERT-HPNE cells.

N2 exhibited stable and non-toxic chemical characteristics upon assays, and its good fluorescence properties allow us to trace the entry of spermine derivatives into cells without cell damage.

### 2.4. Evaluation of Production of Singlet Oxygen of N3 and the Uptake of N3 into PANC-1 Cells

**Figure 3:**
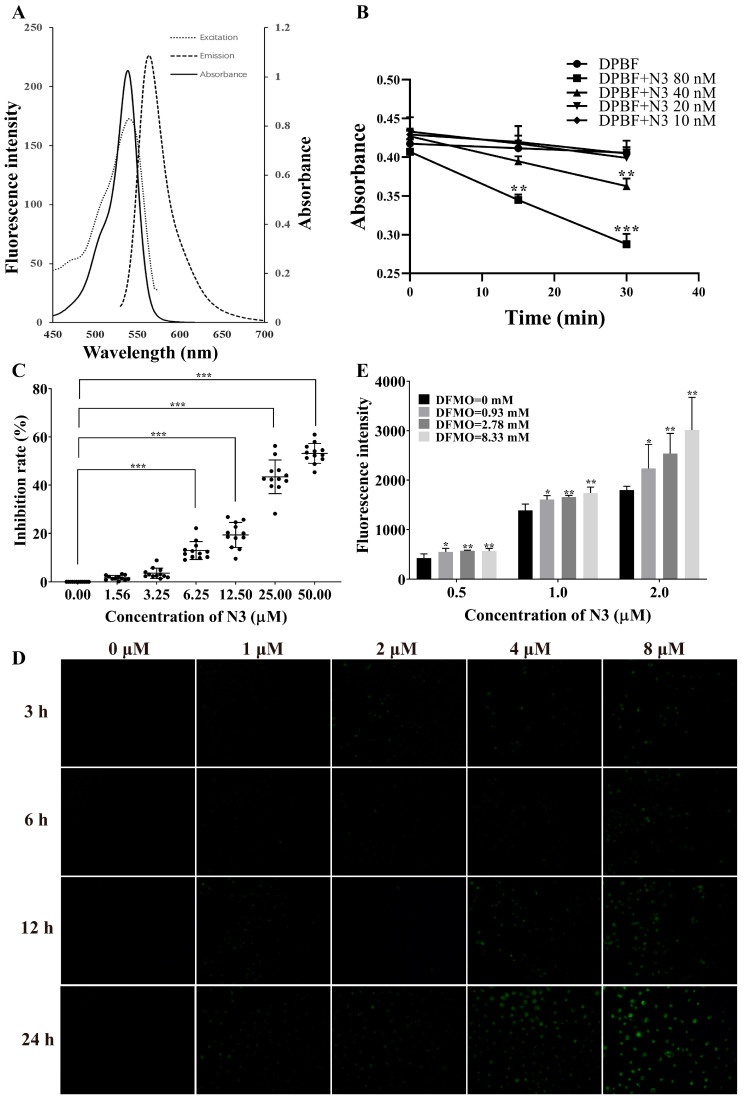
The uptake of N3 into PANC-1 cells through PTS. (**A**) The excitation and emission wavelength of N3 were found at 540 nm and 560 nm; (**B**) the production rates of singlet oxygen by N3 were increased in an irradiation time and concentration-dependent manner; (**C**) evaluation of dark cytotoxicity of N3 in PANC-1 cells; (**D**) the uptake of N3 (0, 1, 2, 4 and 8 µM) into PANC-1 cells for 3, 6, 12 and 24 h (200×); (**E**) evaluation of uptake of N3 into PANC-1 cells using flow cytometry in presence of different concentrations of DFMO (0, 0.93, 2.78 and 8.33 mM). DFMO promoted the uptake of N3 into PANC-1 cells. * *p* < 0.05, ** *p* < 0.01, *** *p* < 0.001.

We synthesized N3 and used it to evaluate the therapeutic effects N3 which is a novel spermine-derived PS that we designed and synthesized, based on BODIPY-2I. The purity and molecular structure of N3 were confirmed by HPLC, MS, and NMR (^1^H NMR and ^19^F NMR) (Supplementary Figure S2). N3 has an excitation wavelength of 540 nm in aqueous solutions, and upon absorbing energy, it mainly generates singlet oxygen. However, N3 can also lose a small part of its energy by emitting faint fluorescence, which has an emission wavelength of 560 nm (Figure 3A). The yield of singlet oxygen is the most important factor for evaluating the photodynamic therapy ability of a PS. Therefore, we first assessed the yield of singlet oxygen by N3 using a DPBF probe (Figure 3B). The attenuation of DPBF absorbance indicates the generation of singlet oxygen. The results showed that at a concentration of 40 nM, N3 had a good yield of singlet oxygen, which was correlated to the dosage of N3 used and the irradiation time.

We subsequently evaluated the dark toxicity of N3 in PANC-1 cells (Figure 3C). The results indicated that the dark toxicity of N3 to PANC-1 cells increased with the dose. However, N3 showed little toxicity to PANC-1 cells when the concentration was below 10 μM, and its dark toxicity could be completely ignored at the nanomolar concentration level. Next, we analyzed the uptake of N3 by PANC-1 cells (Figure 3D,E). The results showed that the fluorescence intensity of N3 absorbed by PANC-1 cells was both time and dose-dependent. Moreover, a low dose of DFMO was also able to promote the uptake of N3 by PANC-1 cells, further confirming that the uptake of N3 by PANC-1 cells is similar to that of N2, being accomplished through the PTS on the PANC-1 cell membrane.

### 2.5. N3 Induced PDT in Pancreatic Cancer Cells

**Figure 4:**
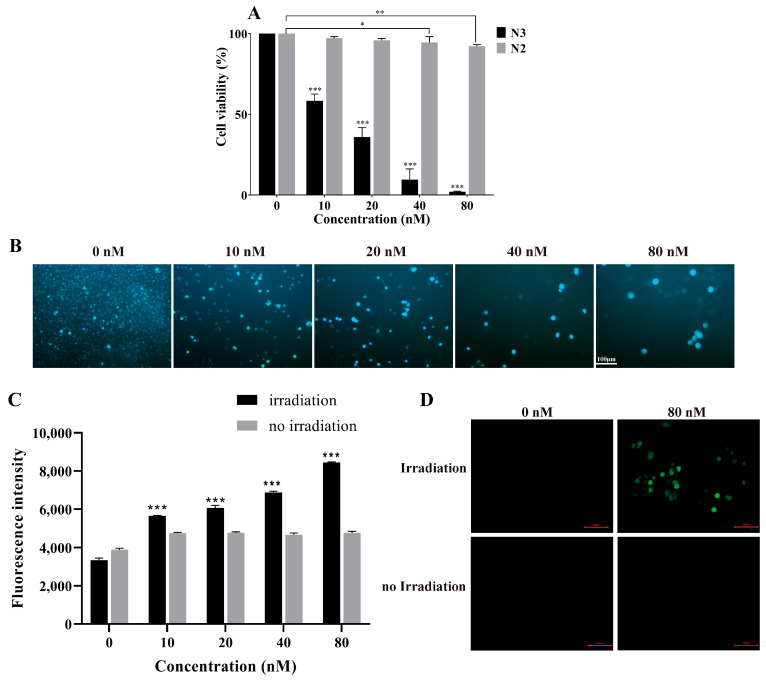
The PDT effect of N3 in PANC-1 cells was in a concentration-dependent manner. (**A**) The PDT was performed in PANC-1 cells with 0, 10, 20, 40, and 80 nM of N3 or N2, N3 exhibited cytotoxicity in a concentration-dependent manner, N2 had no influence on PANC-1 cells, irradiation energy was 6.5 w/m^2^; (**B**) observation of cell nucleus size and cell count of PANC-1 cells with different concentration of N3 using DAPI dye (200×), irradiation energy was 6.5 w/m^2^; (**C**) evaluation of total ROS in PANC-1 cells, irradiation energy was 1.28 w/m^2^; (**D**) observation of ROS content in PANC-1 cells in presence of N3. Green fluorescence was ROS detected by DCFH-DA probe (200×), irradiation energy was 6.5 w/m^2^. Irradiation wavelength: 550 ± 50 nm, * *p* < 0.05, ** *p* < 0.01, *** *p* < 0.001.

To rule out the impact of the heat effect of light on the cells, we first irradiated the PANC-1 cells for 15 or 30 min in the absence of N3. The results indicated that under these experimental conditions, PANC-1 cells were unaffected by light radiation, and no changes in cell morphology were observed (Supplementary Figure S4). Subsequently, we evaluated the PDT effect of N3 (from 10 nM to 80 nM) in PANC-1 and BxPC-3 cells. The results showed that N3 had significant phototoxicity to PANC-1 and BxPC-3 cells, with the survival of PANC-1 and BxPC-3 cells rapidly declining as the N3 concentration increased. At an N3 concentration of 80 nM, 98% of PANC-1 cells (Figure 4A) and 75% of BxPC-3 cells (Supplementary Figure S5A) were killed after irradiation. In contrast, the control group (N2) and N3, without the irradiation group, showed no significant toxicity to PANC-1 and BxPC-3 cells. Moreover, in PANC-1 cells, not only did the number of cells decrease with increasing N3 concentration after irradiation but the cell nuclei also enlarged (Figure 4B). We then examined the effect of N3 on the ROS generation in PANC-1 and BxPC-3 cells. The results showed that after irradiation, ROS in PANC-1 cells significantly increased in an N3 concentration-dependent manner, while there were no significant changes in ROS under non-irradiated conditions (Figure 4C,D). The same phenomenon was observed in BxPC-3 cells (Supplementary Figure S5B).

#### N3-PDT Inhibited the Tumor Growth on the CDX Mouse Model

To evaluate the dosage of N3 for in vivo experiments, acute and chronic toxicity tests were performed in nude mice. In the acute toxicity test, mice were treated with N3 (from 13.97 mg/kg to 111.7 mg/kg) for 24 h to evaluate their survival rate (Supplementary Figure S6A). The results showed that, with the exception of the 13.97 mg/kg of N3 group, the concentrations of 27.93 mg/kg, 55.86 mg/kg, and 111.7 mg/kg of N3 exhibited strong acute toxicity in mice. Therefore, in the subsequent chronic toxicity test, we set the maximum usage concentration of N3 at 6.7 mg/kg to evaluate the treatment concentration for subsequent PDT with N3 (Supplementary Figure S6B,C). The results indicated that apart from the group treated with 6.7 mg/kg of N3, there were no deaths in the other groups within 4 weeks. While mouse weight showed a general downward trend with increasing N3 concentration, there were no significant changes in mouse weight within the first week of treatment. In addition, we confirmed by HE staining pathology that high doses of N3 can damage vital organs in mice, leading to organic lesions, especially in the lungs, liver, and kidneys. (Supplementary Figure S6D).

Subsequently, we repeated the chronic toxicity test of N3 on the PANC-1 cell CDX model. At low doses of N3, the overall tolerance of the CDX model mice to N3 was slightly decreased compared to normal nude mice. However, no significant mouse deaths occurred within the first week of the experiment (Supplementary Figure S7A,B). Furthermore, the experiment also found that without light exposure, N3 had no inhibitory effect on tumor tissue growth (Supplementary Figure S7C–E).

**Figure 5:**
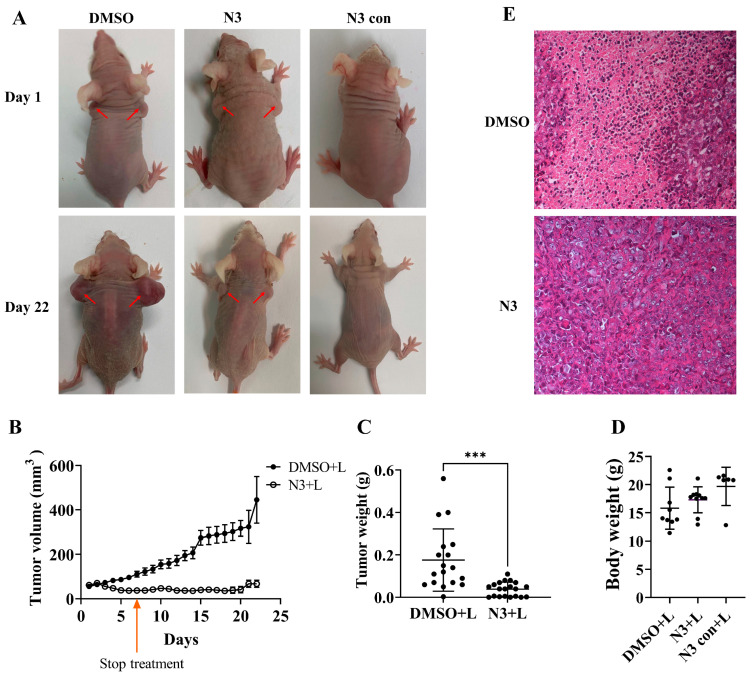
The PDT effect of N3 in CDX mouse model. Mice were divided into the N3 group (tumor-bearing mice, treated with N3 and irradiated every 2 days in the first 7 days), DMSO group (tumor-bearing mice, provided DMSO and irradiated as the N3 group), and N3 control group (normal nude mice without tumor-bearing, treated with N3 and irradiated as the N3 group). (**A**) The tumors on mice in the N3 group and DMSO group on the 1st day and 22nd day. Red arrows indicate tumors (*n* = 10); (**B**) The tumor volume between the N3 group and DMSO group; (**C**) the tumor weight in the N3 group and DMSO group on the 22nd day; (**D**) the body weight of mice in DMSO group, N3 group, and the N3 control group at 22nd day; (**E**) the HE assays of tumors in DMSO group and the N3 group (200×). Irradiation condition: 532 nm, 1.25 w/cm^2^. *** *p* < 0.001.

Finally, we evaluated the PDT effect of N3 in vivo, with N3 used at a dose of 2.23 mg/kg which did not exhibit significant toxicity in the chronic and acute toxicity tests. The results showed that one week after N3-PDT treatment, the tumors in the N3 group had significantly reduced in size (Figure 5A,B). After subsequent observation for two weeks, the tumors in some mice in the N3 group disappeared. On the 22nd day of the experiment, the tumor weights in the N3 group were significantly less than those in the DMSO group (Figure 5C), with no significant differences in mouse body weights among the groups (Figure 5D). The HE staining pathology examination of tumor tissue indicated that the PDT induced by N3 could damage the tumor tissue (Figure 5E). The cells in the tumor tissue of the N3 group were significantly swollen and even ruptured, leading to a loss of cellular structure.

In conclusion, for cancer cells with a high demand for polyamines, such as pancreatic cancer cells, our research indicates that polyamine derivative PSs can be specifically transported into cancer cells through PTS on the cell membrane. After irradiation, these PSs disrupt the redox balance in the cancer cells, leading to a cytotoxic effect. This provides a new strategy and approach for the treatment of cancers that are dependent on polyamine growth.

## 3. Discussion

Cancer is the leading killer affecting human health. A key characteristic of cancer cells is uncontrolled proliferation, and to satisfy this feature, the demand for polyamines in cancer cells is significantly higher compared to normal cells, which includes pancreatic cancer. Pancreatic cancer is a highly malignant tumor with poor treatment outcomes and extremely high mortality rates. Despite significant progress in the treatment of cancers such as lung and liver cancer in recent years, effectively extending the survival period and improving the quality of life for patients, the treatment for pancreatic cancer, whether through surgical or pharmacological means, has not been able to effectively prolong patients’ survival.

In order to meet their own demand for polyamines, cancer cells enhance their ability to synthesize polyamines, thus producing more polyamines for their own needs. On the other hand, cancer cells enhance the activity of the PTS on their cell membrane, thereby increasing their absorption of polyamines and promoting malignant proliferation. Although the nature and molecular structure of the PTS have not been fully elucidated, we understand that the PTS can transport not only natural polyamines but also polyamine derivatives with specific molecular structure requirements. Therefore, we can exploit this feature to transport drugs or PS into cancer cells, thereby killing them. Current studies [29,30,31,32] indicate that the molecules capable of being transported by PTS need at least two positively charged nitrogen atoms, and the optimal distance between nitrogen atoms is about four methylene groups. Furthermore, since spermine has a longer carbon chain and more nitrogen atoms than spermidine, spermine derivatives have a better affinity for PTS than spermidine derivatives. It was reported that the spermine derivative F14512, synthesized based on etoposide structure, can target a variety of cancer cells through the PTS and exert anti-cancer effects [33].

PDT is a technology that uses photosensitive drugs to treat malignant tumors and pathogenic microbial infections, in which PSs are key factors affecting the efficiency of PDT. However, most PSs used clinically lack specificity for cancer cells. Therefore, the discovery of a novel PS with good tumor-targeting abilities and low toxicity will promote the application of PDT.

In this study, we synthesized a spermine-based fluorescent probe (N2) and BODIPY-2I spermine derivative (N3), both of which could target pancreatic cancer cells via PTS. N2 could effectively label and trace PANC-1 pancreatic cancer cells, while N3 could effectively kill PANC-1 and BxPC-3 pancreatic cancer cells under irradiation. Furthermore, we found that the structure of polyamine derivatives could affect the uptake efficiency in PANC-1 pancreatic cancer cells. At the beginning of the N2 design, we hoped it would be synthesized as a fluorescein parent nucleus N1 derived by spermine. However, the synthesized N2 was identified as a symmetrical compound with two N1 derived by a spermine. Despite its structure not conforming to our expectations, experimental verification showed that N2 could still be transported into PANC-1 cells via PTS. Accordingly, we analyzed the distance between each nitrogen atom in the N2 structure (Supplementary Figure S8), and found that the distance between two nitrogen atoms on 4-diaminomethyl-1,8-naphthalic anhydride (N1) is approximately 6.500 Å, which is close to the length of four methylene groups in spermine (6.726 Å). This result indicates that the distance between nitrogen atoms is crucial for PTS and nitrogen atoms do not necessarily have to be on the aliphatic carbon chain, they can be on the ring structure.

DFMO is a polyamine biosynthesis inhibitor that causes rapid depletion of polyamines in cancer cells, leading to a halt in cell growth, and was used in early cancer treatments [34]. However, the high concentration required for DFMO in clinical treatments leads to substantial side effects, and this therapeutic effect can be easily evaded by cancer cells by enhancing the activity of PTS for the uptake of exogenous polyamines, thus DFMO has not become a clinical drug for cancer treatment [35]. Consequently, DFMO has not yet transcended the realm of early-phase clinical trials [36,37,38,39], which are sparse and date back several decades. Due to the unlimited proliferation characteristic of cancer cells, their demand for polyamines significantly increases, and PTS is highly expressed in many cancer cells, including pancreatic cancer. Therefore, we hypothesize that DFMO can assist cancer cells in promoting the uptake of polyamine derivatives. In this study, we confirmed that PANC-1 cells can accumulate more N2 and N3 after treatment with a low dose of DFMO. This result indicates that for the treatment of cancer with polyamine derivative drugs, combining with a low dose of DFMO in clinical practice may achieve better therapeutic effects.

Polyamine metabolism is associated with the oncogene *MYC*, which drives cells to increase the expression and activity of ODC, promoting the proliferation of cancers related to dysregulated polyamine metabolism [40,41] (including leukemia [42,43], lung cancer [44], neuroblastoma [44,45,46], and breast cancer [47]). In addition, many other proliferative signaling pathways or proto-oncogenes also regulate polyamine metabolism, promoting tumor cell proliferation through stimulating polyamine biosynthesis or exogenous uptake, such as BRAF mutant melanoma, hepatocellular carcinoma, colon cancer, prostate cancer, and neuroblastoma. Therefore, we hypothesize that N3 may also have therapeutic effects on these cancer cells, as their proliferation also depends on high levels of polyamines within the cell.

In this study, we also evaluated the in vivo efficacy of N3-mediated PDT. In a mouse model of pancreatic cancer with PANC-1 cell xenografts, significant tumor reduction was observed one week after N3-PDT treatment, with the dosage of N3 used being only 2.23 mg/kg, yielding no apparent dark toxicity. Unlike traditional drug treatments, in PDT, the concentration of PS can be further reduced, and the light intensity or exposure time increases, thus reducing the toxic side effects of PS without affecting the therapeutic efficacy. Moreover, our results also demonstrated the in vivo targeting specificity of N3 towards PANC-1 cells. It is well-known that skin damage is a severe side effect of PDT treatment. In our experiment, mice injected with DMSO exhibited no therapeutic effects on tumors and no skin damage under light exposure, suggesting that the light energy used in this study was harmless to mouse skin. However, upon light exposure, the injection of N3 suppressed tumor growth significantly and was accompanied by skin damage. Interestingly, when the same dose of N3 was administered to normal nude mice (N3 control group), skin damage was much milder compared to the N3 group. According to the histological depth assessment for burn wound grading [48,49,50,51,52,53], Class-III (eschar) burns represent the most severe type. One week post-PDT, the incidence of Class-III skin damage in the N3 control group was lower than in the N3 group (Table 1). This indicates that N3 accumulated in the pancreatic cancer tissue, leading to more severe skin damage. One week after stopping PDT, the damaged skin of the mice in the N3 group was gradually replaced by normal skin, and complete recovery was achieved after two weeks.

While this study has made some progress, it also has certain limitations. First, the work only utilized pancreatic cancer cells as the object of study and did not encompass other types of cancer. Considering the heterogeneity and complexity of cancer, future studies should expand the experimental scope to further validate the broad applicability of this strategy. Second, we used a xenograft mouse model rather than an orthotopic mouse model. Although the xenograft model is easy to manipulate and observe and there are no obstacles in the penetration of excitation light compared with the orthotopic model, there may be limitations in simulating the tumor microenvironment and tumor biological characteristics in humans. Furthermore, although the use of PS shows potential for the treatment of drug-resistant malignant tumors, we still recognize that PDT cannot completely replace existing cancer treatment methods. Instead, it mainly serves as an effective supplement to current treatments.

When deliberating on strategies for photodynamic therapy (PDT) of pancreatic cancer, the selection of the light source is of paramount importance. Although light within the 550 ± 50 nm wavelength range is considered for PDT due to its alignment with the absorption spectra of certain photosensitizers, it is imperative to acknowledge that this range exhibits relatively poor penetration through skin tissues. Scholars have reported that light around 550 nm encounters limited penetration in human skin tissues [54,55,56], primarily attributed to the elevated absorption coefficients of oxyhemoglobin and water at this wavelength. This limitation may impede the efficacy of PDT, as light must traverse the skin and other tissue layers to reach the pancreatic tumor. Increased absorption and scattering of 550 ± 50 nm light by skin tissues could lead to a reduction in the light intensity at the tumor site, thereby diminishing the excitation efficiency of the photosensitizer and the generation of reactive oxygen species (ROS), which are crucial for the cytotoxic effect in PDT [4,7,8,17,57,58,59]. To surmount this challenge, researchers are exploring various tactics. For instance, the selection of photosensitizers with higher tissue penetration or optimization of the light source’s power and exposure duration can enhance the delivery of light. Moreover, the integration of advanced drug delivery systems, such as nanoparticle carriers, can augment the concentration of photosensitizers at the tumor site, facilitating effective PDT even at lower light intensities [6,7,60]. Additionally, it is necessary to contemplate enhancing the light penetration by improving the design of the light source and photosensitizer. Utilizing light sources with longer wavelengths may increase the depth of tissue penetration, although this could necessitate chemical modifications to existing photosensitizers to ensure their effective light absorption and ROS generation at the new wavelengths [10,61]. Well, while the limited skin penetration of 550 ± 50 nm light poses a challenge to the efficacy of PDT for pancreatic cancer, a comprehensive approach considering the selection of photosensitizers, optimization of light sources, and refinement of drug delivery systems holds promise to overcome these obstacles and enhance the clinical outcomes of PDT in treating pancreatic cancer.

In summary, our study suggests that using polyamine derivatives as PS to treat cancer is a novel therapeutic strategy. The PTS on the cancer cell membrane can selectively transport these PSs into cancer cells, which are then killed by localized irradiation. In subsequent studies, we will optimize the molecular structure of N3 to make its excitation wavelength in the near-infrared region, thereby achieving better penetration in deep tissues and maximizing its role in the entire cancer treatment regimen.

## 4. Materials and Methods

### 4.1. Synthesis of N1, N2, and N3

The synthetic route of N1 and N2 was illustrated in Scheme 1A,B.

Synthesis of N1 (Scheme 1A): 4-Br-1,8-naphthalic anhydride (0.90 mmol, 3.00 g), dimethylamine (54.3 mmol, 2.40 g), and catalytic amount CuI were added to 8 mL of dimethylformamide (DMF). The reaction mixture was refluxed under a nitrogen atmosphere for 12 h. When the reaction finished, the hot reaction mixture solution was poured into cold water (400 mL), and the precipitated solid was filtrated with a Buchner funnel and then washed with ethanol. Finally, the yellow product was dried in a vacuum oven and used for the next reaction without further purification.

Synthesis of N2 (Scheme 1B): N1 (1.98 mmol, 0.48 g) was added to the mixture of ethanol (30.0 mL) and DMF (10.0 mL). The reaction mixture was heated to reflux and then triethylamine (5.0 mL) was added. When the N1 solid was completely dissolved, the spermine (1.98 mmol, 0.40 g) in ethanol was slowly added into the reaction system. The reaction mixture was refluxed under a nitrogen atmosphere for 12 h. When the reaction finished, ethanol was removed by vacuum evaporation, and the residue was poured into water (400 mL), providing a yellow solid. The solid was filtrated with a Buchner funnel, washed with ethanol, and then dried by vacuum. ^1^H NMR (400 MHz, CDCl_3_), δ 8.55 (dd, *J* = 7.3, 0.9 Hz, 1H), 8.45 (d, *J* = 8.2 Hz, 1H), 8.41 (dd, *J* = 8.5, 0.9 Hz, 1H), 7.64 (dd, *J* = 8.3, 7.4 Hz, 1H), 7.10 (d, *J* = 8.2 Hz, 1H), 4.23 (t, *J* = 6.9 Hz, 2H), 2.68 (t, *J* = 6.8 Hz, 2H), 2.61 (d, *J* = 5.7 Hz, 2H), 1.93 (dt, *J* = 29.7, 11.3 Hz, 4H), 1.28 (s, 10H), and 0.85 (dd, *J* = 12.3, 8.7 Hz, 6H). [13]C NMR (101 MHz, CDCl_3_) δ 164.71, 164.17, 156.89, 132.68, 131.18, 131.03, 130.16, 125.28, 124.89, 113.35, 113.22, 49.77, 48.38, 47.03, 44.81, 43.56, 38.03, 37.12, 29.74, 28.39, and 27.85. HRMS calc for C_38_H_44_N_6_O_4_ [M + H]^+^ 649.3502, found 649.3480.

The synthetic route of PS bearing spermine (N3) was illustrated in Scheme 1C.

① Preparation of compound **2**

4-carboxybenzaldehyde (33.3 mmol, 5.0 g) and 2,4-dimethyl pyrrole (74.3 mmol, 7.7 mL) were dissolved in dichloromethane (350 mL) in a 1000 mL round bottom flask under N_2_. Then two drops of trifluoroacetic acid (TFA) were added and the solution was stirred under N_2_ for 17 h at room temperature. After the addition of 2,3-Dichloro-5,6-dicyano-1,4-benzoquinone (DDQ, 33.3 mmol, 7.56 g) to the reaction mixture, stirring was continued for another 4 h. Then Et_3_N (76 mL) and BF_3_⋅OEt_2_ (76 mL) were successively added. After 12 h, the reaction mixture was quenched with water (500 mL) and then extracted by dichloromethane. After drying over anhydrous Na_2_SO_4_, the solvent was evaporated, and the residue was purified by silica gel column chromatography (EA:PE = 0~40%) to provide a red solid (yield 12%).

② Preparation of compound **3**

Compound 2 (2.43 mmol, 0.90 g), EtOH (90 mL), and iodine (6.00 mmol, 1.50 g) were added into a 250 mL round-bottom flask, followed by the addition of iodic acid (4.89 mmol, 0.9 g) in water (3.0 mL). The reaction mixture was stirred at 60 °C for 1 h, then the solvent was evaporated, and the residue was purified by silica gel column chromatography (EA:PE = 0~40%) to provide a deep red solid (yield 40%).

③ Preparation of N3

Compound 3 (0.61 g, 0.98 mmol), N,N′-succinimide carbonate (DSC, 0.30 g, 1.18 mmol), N,N-diisopropylethylamine (DIPEA, 0.25 g, 2 mmol), and spermine (0.20 g, 0.98 mmol) were dissolved in dichloromethane (50 mL), and the mixture was stirred at room temperature for 5 h. After removing the solvent by vacuum evaporation, the residue was purified by prep-HPLC (TFA method) to provide a red solid (yield 64%). ^1^H NMR (400 MHz, DMSO-d6): δ 8.88 (t, *J* = 5.2, 10.8 Hz, 1H), 8.73 (brs, 2H), 8.58 (brs, 2H), 8.07 (d, *J* = 8 Hz, 2H), 7.89 (brs, 3H), 7.55 (d, *J* = 8.4 Hz, 2H), 3.38 (m, 2H), 2.92 (m, 10 H), 2.55 (s, 6 H), 1.89 (m, 4H), 1.63 (m, 4H), 1.33 (s, 6 H). [19]F NMR (376 MHz, CDCl_3_): δ −143.12, 143.20, 143.28, 143.37. LC-MS: *m*/*z* 805.2 [M + H]^+^.

#### 4.1.1. Determination of Photophysical Properties of Compounds

The absorption spectrum of the compounds (N1, N2, and N3) was measured at 10 µM using an Ultraviolet spectrophotometer (UV-2600, Shimadzu, Kyoto, Japan). Fluorescence and excitation spectra were obtained using a Fluorescence spectrometer (F-4600, HITACHI, Tokyo, Japan). The stock solution of compounds N1 and N2 was dissolved in absolute ethanol and N3 in DMSO. All experiments were performed in a quartz cell at room temperature with scanning steps of 1 nm. In order to detect the fluorescence properties of N2 in water and organic solvent, N2 was dissolved in ethanol, dimethyl sulfoxide (DMSO), DMEM, and water at 4 μM. The fluorescent signal was detected by a fluorescence microscope (DMI3000B, Leica, Wetzlar, Germany).

#### 4.1.2. Cell Cultures

The pancreatic cancer cell line PANC-1, BxPC-3, and normal human pancreatic ductal epithelial cell line hTERT-HPNE were purchased from Shanghai Zhong Qiao Xin Zhou Biotechnology Co., Ltd. (Shanghai, China). These cells were cultured in DMEM (Gibco, Waltham, MA, USA) containing 10% fetal bovine serum (Gibco, Grand Island, NY, USA) and 1% penicillin-streptomycin mixture (Biosharp, Hefei, China), incubated with 5% CO_2_ at 37 °C.

#### 4.1.3. Cytotoxicity Detected with Cell Counting Kit 8

The cytotoxicity of N2, DMFO, and N3 was evaluated using a cell counting kit-8 (CCK-8 kit, Dojindo Molecular Technologies Inc., Kumamoto, Japan) according to the manufacturer’s instructions. The cells were seeded in a 96-well plate at 6 × 10^3^ cells/well and cultured for 24 h. Then, the medium was replaced with DMEM containing different concentrations of N2 (from 1.56 μM to 50 μM), DFMO (from 1.56 mM to 50 mM), or N3 (from 1.56 µM to 50 μM) and incubated for 24 h. Cells were washed thrice with ice-cold PBS and cultured in fresh DMEM. CCK-8 solution was subsequently added into each well and incubated at 37 °C for 2 h. The absorbance at 450 nm was measured using a spectrophotometer (Synergy H1, Biotek, Winooski, VM, USA).

#### 4.1.4. The Uptake of N1, N2, and N3 into Cells Observed Using Fluorescence Microscope

The cells were seeded into 24-well plates at 4 × 10^4^ cells/well and cultured for 24 h. Then, the medium was replaced with DMEM containing different concentrations of N1 (1 and 2 μM), N2 (1 and 2 μM), or N3 (1, 2, 4, and 8 μM) and incubated for 3, 6, 12, and 24 h. Fluorescent signals were observed using a fluorescence microscope (DMI3000B, Leica, Wetzlar, Germany).

The cells were seeded into 24-well plate at 4 × 10^4^ cells/well and cultured for 24 h. Then, the medium was replaced with DMEM containing different concentrations of DFMO (0, 0.31, 0.93, and 2.78 mM) and incubated for 12 h. Subsequently, the medium was replaced with DMEM containing different concentrations of DFMO and N1 (1 and 2 μM) or N2 (1 and 2 μM) and incubated for another 12 h. The fluorescent signal was observed using a fluorescence microscope (DMI3000B, Leica).

#### 4.1.5. The Uptake of N2 into Cells Evaluated Using Flow Cytometry

The cells were seeded into 24-well plates at 4 × 10^4^ cells/well and cultured for 24 h. Then, the medium was replaced with DMEM containing different concentrations of N2 (from 0 μM to 40 μM) and incubated for 8 h. Subsequently, cells were washed with PBS and collected, and the fluorescent signal was evaluated using a flow cytometry (BD Biosciences, Franklin Lakes, NJ, USA).

#### 4.1.6. The Uptake of N2 and N3 into Cells Evaluated Using Spectrofluorometry

The cells were seeded into a 96-well plate at 6 × 10^3^ cells/well and cultured for 24 h. After which, the medium was replaced with DMEM containing different concentrations of DFMO (0, 0.31, 0.93, and 2.78 mM) and incubated for 12 h. The medium was later replaced again with DMEM containing different concentrations of DFMO and N2 (2 μM) and then incubated for an additional 12 h. Alternatively, the medium was replaced with DMEM containing different concentrations of DFMO (0, 0.93, 2.78, and 8.33 mM) and incubated for 12 h, followed by a replacement with DMEM containing different concentrations of DFMO and N3 (0.5, 1, and 2 μM) for another 12 h incubation. The fluorescence intensity was determined using a spectrofluorometer (FC microplate reader, Thermo Fisher Scientific, Waltham, MA, USA).

#### 4.1.7. Competition Assay Evaluated Using Spectrofluorometry

The cells were collected into 1.5 mL EP tubes (4 × 10^4^ cells/tube). N2 mixed with different polyamines (putrescine, spermidine, spermine, and mixtures of putrescine, spermidine, and spermine) was added; the final concentration of N2 was 2 μM and polyamines or mixture of polyamines were 10 μM. Subsequently, 100 μL of cell culture in a tube was transferred into a 96-well plate, respectively, the fluorescence change of each tube was recorded using a spectrofluorometer (FC microplate reader, Thermo Fisher Scientific, USA) during 200 min.

#### 4.1.8. Productivity of Singlet Oxygen Evaluated Using Spectrofluorometry

N3 was added into a 96-well plate with a final concentration of 0, 10, 20, 40, and 80 nM, then irradiated under light (550 ± 50 nm, 6.5 w/m^2^) for 0, 15, and 30 min. The productivity of singlet oxygen was evaluated using 1,3-diphenylisobenzofuran (DPBF) probe following the manufacturer’s instruction (Macklin, Shanghai, China). The absorbance at 415 nm was measured using a spectrofluorometer (FC microplate reader, Thermo Fisher Scientific, USA).

#### 4.1.9. Phototoxicity in Cells

The cells were seeded into a 96-well plate at 6 × 10^3^ cells/well and cultured for 24 h. Then, the medium was replaced with DMEM containing 0, 10, 20, 40, and 80 nM of N2 or N3 and incubated for 12 h in the dark. Subsequently, the cells were irradiated (450 ± 50 nm, 6.5 w/m^2^ for N2; 550 ± 50 nm, 6.5 w/m^2^ for N3) for 15 or 30 min. After incubation in the dark for another 12 h, cells were washed with PBS, and a CCK-8 assay was performed to evaluate cell viability.

The cells were seeded into a 24-well plate at 4 × 10^4^ cells/well and cultured for 24 h. Then, the medium was replaced with DMEM containing 0, 10, 20, 40, and 80 nM of N3 and incubated for 12 h in the dark. Subsequently, the cells were irradiated (550 ± 50 nm, 6.5 w/m^2^) for 30 min. After incubation in the dark for another 12 h, cells were washed with PBS, and the cell nucleus size and cell count were observed with DAPI dye.

#### 4.1.10. Evaluation of ROS in Cells

The cells were seeded into a 96-well plate at 6 × 10^3^ cells/well and cultured for 24 h. Then, the medium was replaced with DMEM containing 0, 10, 20, 40, and 80 nM of N3 and incubated for 12 h in the dark. Subsequently, the cells were irradiated (550 ± 50 nm, 1.28 w/m^2^) for 30 min. After incubation in the dark for another 12 h, the medium was replaced, and the content of ROS in cells was evaluated using 2,7-Dichlorodihydrofluorescein diacetate (DCFH-DA, Reactive Oxygen Species Assay Kit, Solarbio, Beijing, China) probe with excitation and emission wavelength at 488 nm and 525 nm. The fluorescence intensity was measured using a spectrofluorometer (FC microplate reader, Thermo Fisher Scientific, USA).

#### 4.1.11. Animals

Nude mice (4-week-old, 16–18 g) were obtained from Beijing Huafukang Bioscience Co., Inc., (Beijing, China) for an in vivo experiment. The experimental procedures were conducted in accordance with the Ethics Committee for Experiments on Animals of Laboratory Animal Center of China Three Gorges University.

#### 4.1.12. Acute and Chronic Toxicity of N3 In Vivo

For the acute toxicity test of N3, nude mice were randomly divided into several groups by gender, then intraperitoneal injected with 50 μL of DMSO or different concentrations of N3 (from 13.97 mg/kg to 111.7 mg/kg). DMSO and N3 were given once for one-day toxicity observation.

For chronic toxicity test of N3, different concentrations of N3 (from 0.42 mg/kg to 6.7 mg/kg) were given every 2 days for a 30-day observation, and the behaviors of each mouse were recorded every 2 days. After observation, the mice were sacrificed, and the tissues of the heart, liver, spleen, lung, and kidney were obtained for HE assays.

#### 4.1.13. Cell-Derived Xenograft Mouse Model of Pancreatic Cancer

To obtain subcutaneous xenografts, cultured 2 × 10^6^ cells were collected and injected into both sides of the scapula of nude mice to establish the cell-derived xenograft (CDX) mouse model. The solid tumors were transplanted from nude mouse to nude mouse.

#### 4.1.14. The Dark Toxicity of N3 on CDX Mouse Model

The tumors were transplanted at both sides of the scapula of nude mice. When the tumor volume reached 55–65 mm^3^, mice were randomly assigned to the control group, 1.68 mg/kg N3 group, and 3.35 mg/kg N3 group, with seven tumor-bearing mice in each group. DMSO or N3 were intraperitoneally injected into mice every 2 days and kept in the dark. After observation, the mice were euthanized, and the tissues of the tumor were obtained for HE assays.

#### 4.1.15. The Phototoxicity of N3 on CDX Mouse Model

The tumors were transplanted at both sides of the scapula of nude mice. When the tumor volume reached 55–65 mm^3^, the mice were randomly divided into 2 groups as DMSO group and the N3 group (2.23 mg/kg), consisting of 10 tumor-bearing mice in each group. DMSO and N3 were intraperitoneal injected into the mice every 2 days in the first 7 days. 2 h after injection, the irradiation (532 nm, 1.25 w/cm^2^) on the tumor was performed for 2 min. Then, the mice were observed for another 14 days. In addition, a N3 control group in normal nude mice without tumors was performed as well as the N3 group. After observation, the mice were sacrificed, and tumor tissues were obtained for HE assays.

#### 4.1.16. Molecular Structure Analysis of N2

Geometry optimized molecular structure of N2 was determined by density functional theory (B3LYP) with 6-31G (d,p) basis sets. The distances between neighboring nitrogen atoms were evaluated.

#### 4.1.17. Statistical Analysis

All of the in vitro experiments were conducted independently at least three times. Statistical analyses were performed using SPSS Statistics v18.0. The data present was mean ± s.d. The *p* value was calculated by the Student’s *t*-test.

## Figures and Tables

**Table 1:** The classification of skin burn injuries in each group by N3-PDT. The rate of skin burn injury in each group was displayed. The burn wounds were classified as Class-III (eschar burns), Class-II (blister burns), and Class-I (erythema burns) types.

Group	Drug	Tumor	N3-PDT 1st (%)		N3-PDT 2nd (%)		N3-PDT 3rd (%)	
			III	II	III	II	III	II
N3	N3	+	70	30	20	80	11.1	88.9
N3 con	N3	−	100	0	100	0	33.3	66.7
DMSO	DMSO	+	0	0	0	0	0	0

## Data Availability

The original contributions presented in the study are included in the article/Supplementary Material, further inquiries can be directed to the corresponding author/s.

## Supplementary Materials

The following supporting information can be downloaded at: https://www.mdpi.com/article/10.3390/molecules29174277/s1.
