# Supplementary material for: Polyamine Derived Photosensitizer: A Novel Approach for Photodynamic Therapy of Cancer
Source: Molecules. 2024 Sep 9;29(17):4277. doi: 10.3390/molecules29174277 (PMC11397399; doi:10.3390/molecules29174277)
Supplement: Supplementary file 1 [file molecules-29-04277-s001.zip › molecules-3029060-supplementary.pdf]

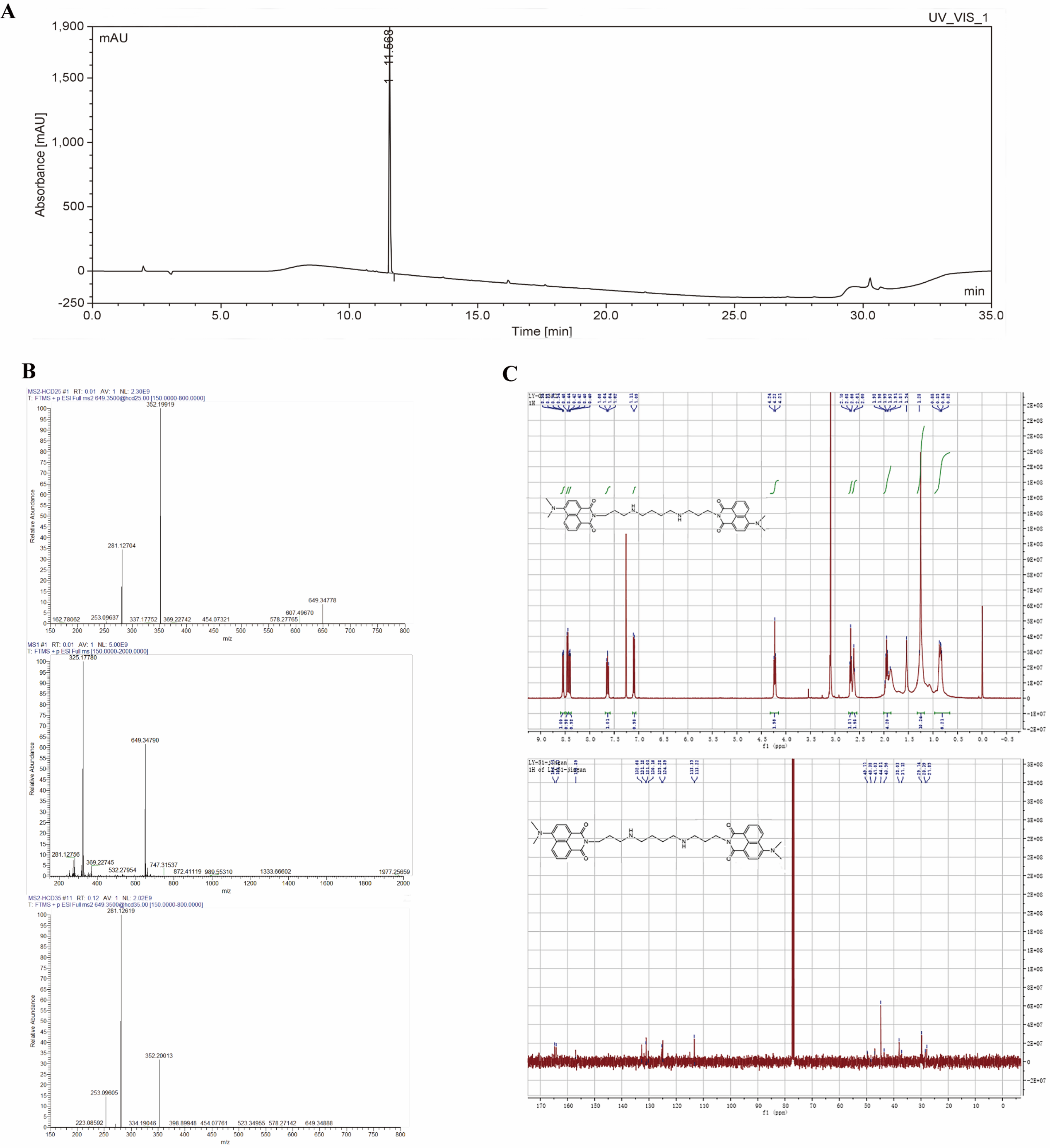

Supplementary Figure S1. Structural identification of Spermine-based fluorescent probe (N2). (A) Purification of N2 by High Performance Liquid Chromatography (HPLC); (B) Analysis of N2 by HPLC-MS; (C) Analysis of N2 by NMR (^{1}H NMR and ^{13}C NMR).

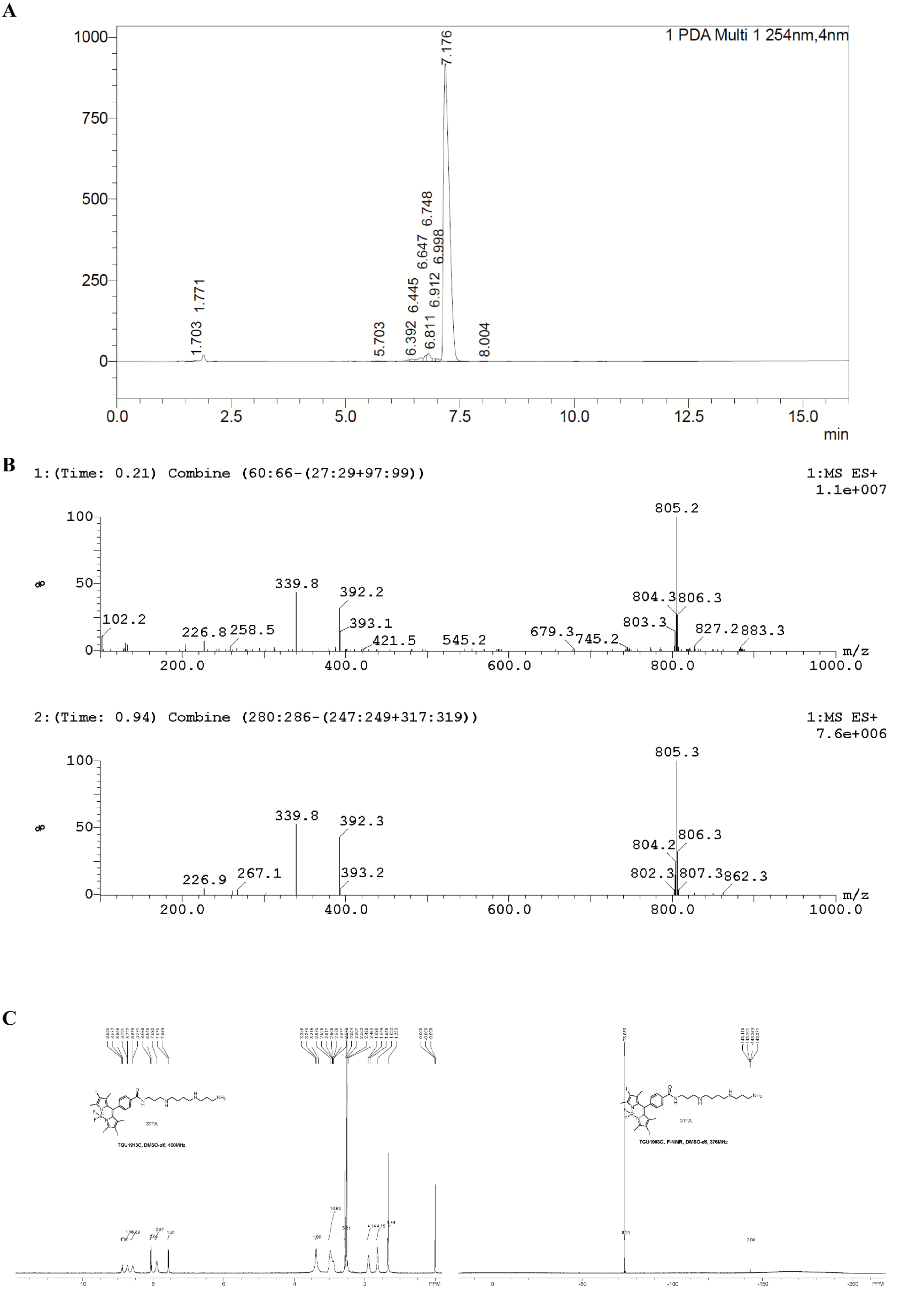

Supplementary Figure S2. Structural identification of photosensitizer spermine derivatives (N3). (A) Purification of N3 by HPLC; (B) Analysis of N3 by HPLC-MS; (C) Analysis of N3 by NMR (^{1}H NMR and ^{19}F NMR)

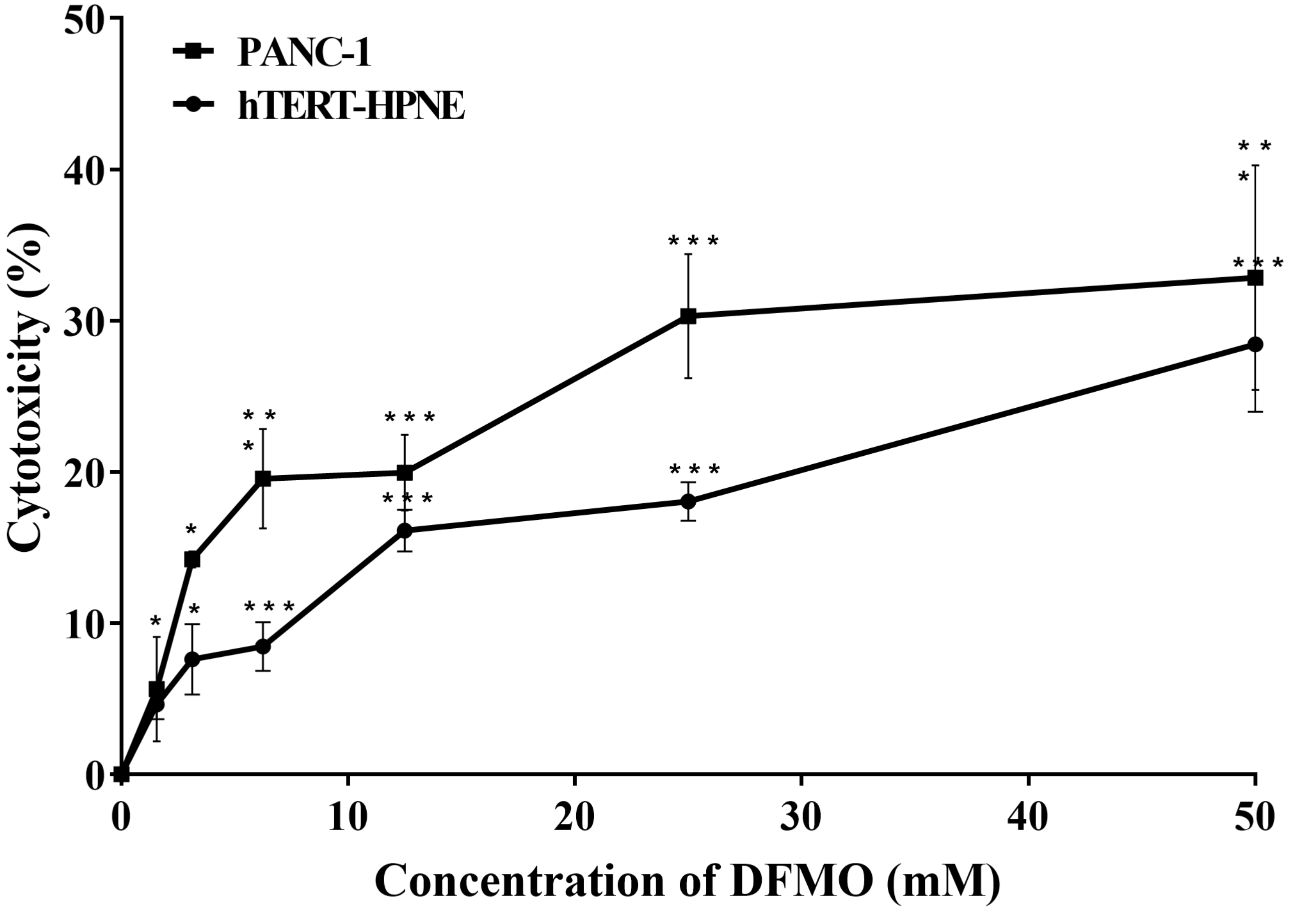

Supplementary Figure S3. The cytotoxicity of DFMO in PANC-1 and hTERT-HPNE cells. The final concentration of DFMO was from 50 mM to 1.56 mM. *$P<0.05$, **$P<0.01$, ***$P<0.001$.

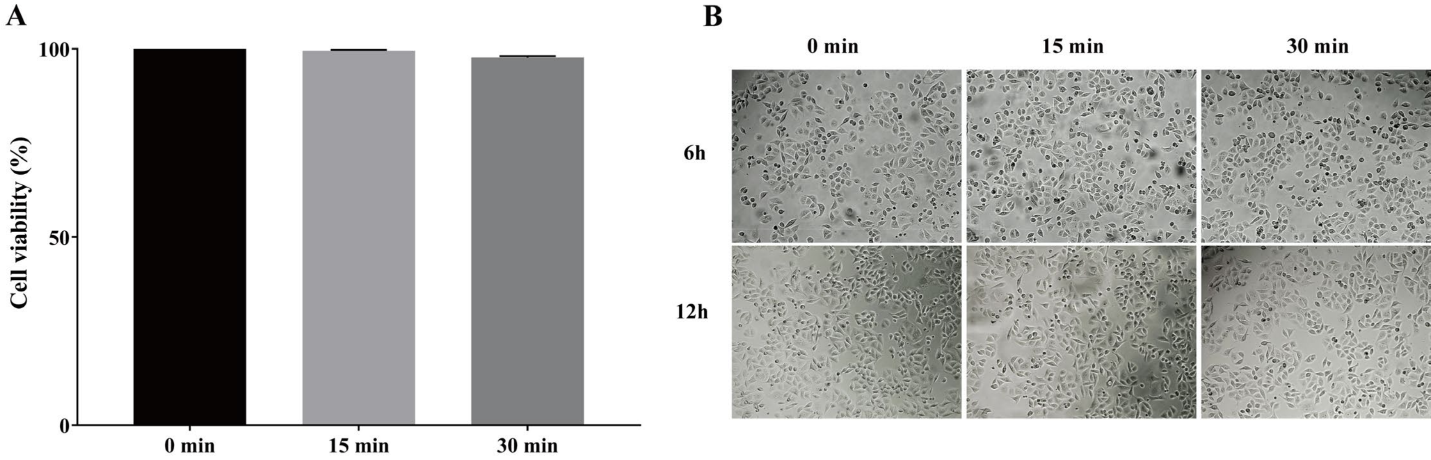

Supplementary Figure S4. The effect of irradiation only in PANC-1 cells. (A) The cell proliferation of PANC-1 cells after different irradiation time; (B) The cell morphology of PANC-1 cells after different irradiation time (100×).

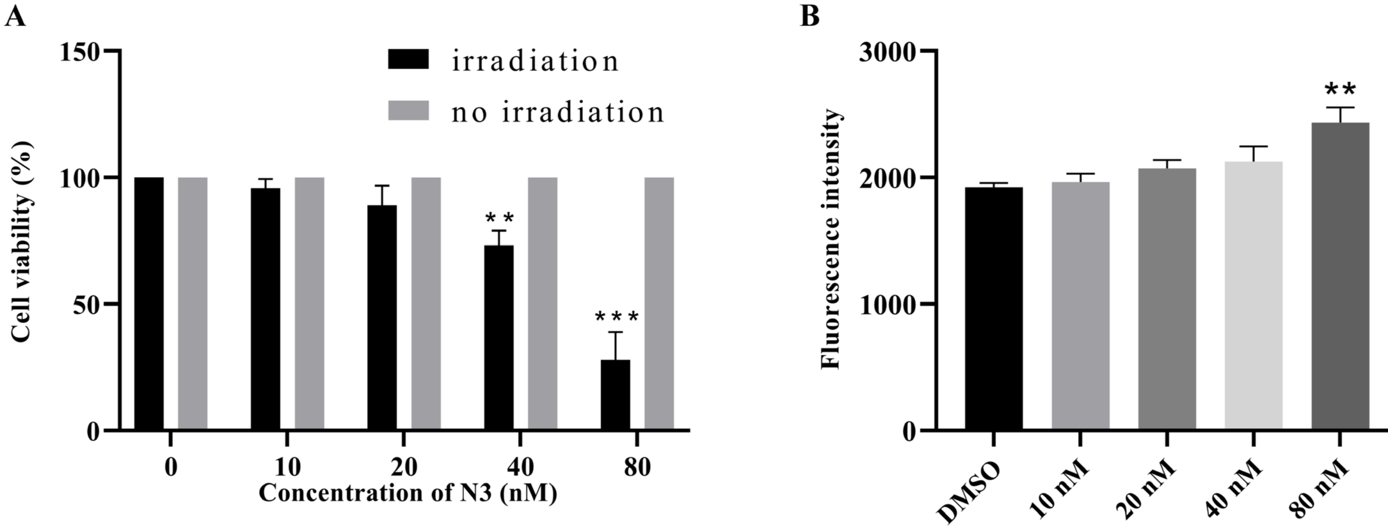

Supplementary Figure S5. The PDT effect by N3 in Bx-PC3 cells. (A) The PDT effect in Bx-PC3 cells with 0, 10, 20, 40 and 80 nM of N3 after irradiation. (B) The ROS generation in Bx-PC3 cells using different concentrations of N3. **P < 0.01, ***P < 0.001.

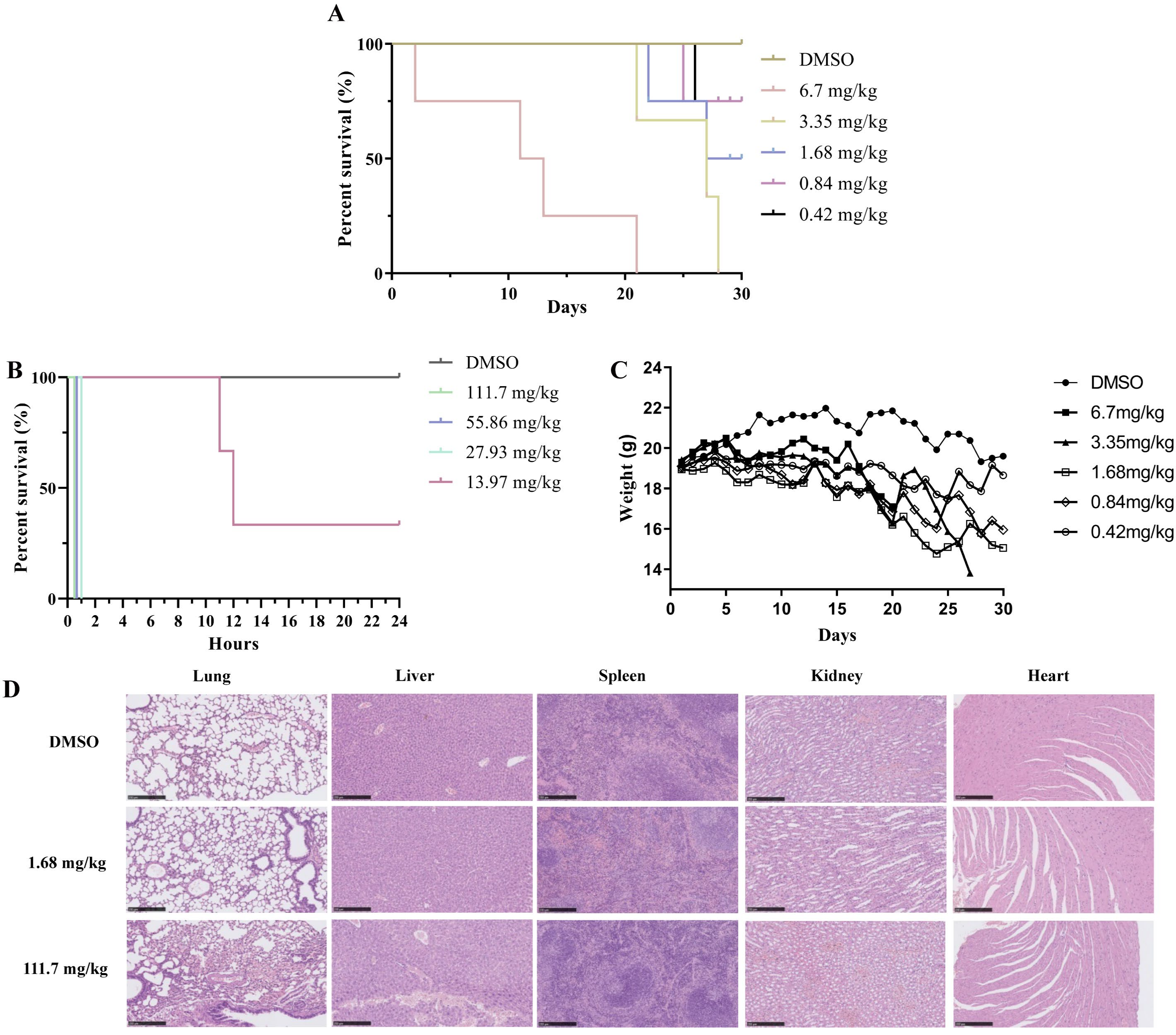

Supplementary Figure S6. The dark toxicity of N3 in normal nude mouse. (A) The survival rate of mouse in acute toxicity test of N3 without irradiation in normal nude mouse (n=6); (B) The survival rate of mouse in chronic toxicity test of N3 without irradiation in normal nude mouse (n=6); (C) The body weight of normal mouse after a 30-day intraperitoneal injection in chronic toxicity test; (D) The HE assays of important organs in normal nude mouse with different treatment concentration of N3 (100×).

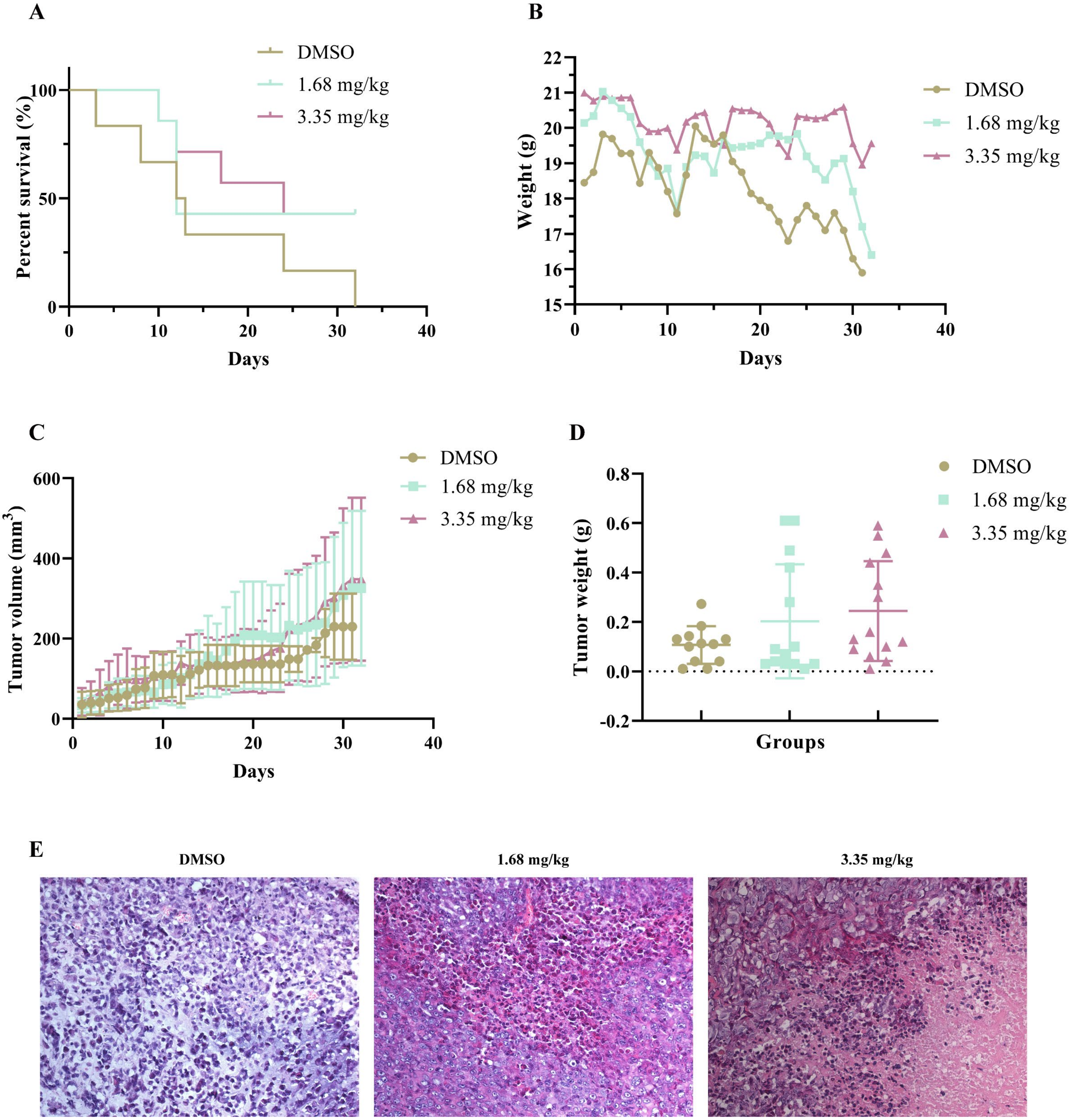

Supplementary Figure S7. The dark toxicity of N3 on CDX mouse model.The survival rate of mouse(A),The tumor weight (B), tumor size (C) and body weight (D) of CDX mouse model after 30-day intraperitoneal injection of N3 (n=7); (E)The HE assays of tumors in CDX mouse model with different treatment concentration of N3 in dark (100×).

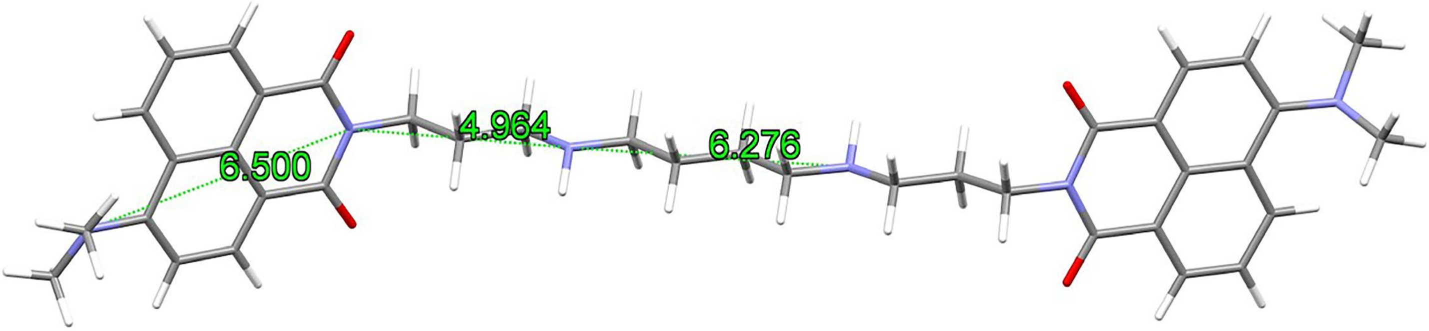

Supplementary Figure S8. The distance between nitrogen atoms in molecular structure of N2.
